# Mr. Millet, on Gout

**Published:** 1803-09-01

**Authors:** R. O. Millett

**Affiliations:** Hayle, Cornwall


### *To the Editors of the Medical and Physical Journal.*

GENTLEMEN,

SEVERAL instances of the beneficial effects of reduced temperature for the removal of gouty complaints, which have appeared in the Medical and Physical Journal, and Dr. Kinglake's request to medical men, to make known their intelligence on the subject, have induced me to communicate the following case.

Moses

Moses Simons, aged 54, accustomed to a very liberal use of spirituous liquors, had been afflicted with gout generally three or four times a year for ten years, sometimes in his hands, at other times in his knees, but his ancles and toes chiefly suffered; when those parts were attacked they swelled, inflamed, and had a glossy appearance, attended with a burning sensation, sometimes his head and stomach were also affected with pains, and the latter with nausea and dyspepsia.

About two years ago, when his toes happened to be affected with gout, a neighbour of his, (not a medical man) called to see him, and advised him to hold his foot under a cock of cold water; by the help of a stick he with difficulty walked about 100 yards, and kept his foot for ten minutes under a large cock, from which cold water was poured with great force, during which time he heard a crackling noise issuing from his foot, and found such immediate relief, that he threw aside his stick and walked to his house with ease, and has not since had any return of gout, or symptoms of it, unless an extensive livid coloured inflammation with very little tumour, which took place a year ago in his left hypochondriac region, may be considered as of that nature. As this inflammation remained stationary several days, he sent for me; I thought it might not suppurate, and after he had taken some laxatives it subsided without disquamation of the cuticle; a fortnight afterwards another inflammation took place in his arm, which soon became hard and pointed as a phlegmon; warm poultices were applied and it suppurated, forming several deep holes, which as soon as he could be prevailed upon, I laid open into one wound, and it healed in two months time; the use of his arm is quite restored and he is very well in every respect, and continues in his employment at the copper-works, where he has been for ten years exposed to great heat, and the sudden application of cold when in perspiration, and inhaling the most noisome effluvia proceeding from the furnace.

I am, &c.

R. O. MILLETT, Jun.

*Hayle, Cornwall, July 28, 1803.*

